# Pleuritic Chest Pain in a Young Female: A Reminder for Acute Health Care Providers

**DOI:** 10.1155/2014/824786

**Published:** 2014-08-27

**Authors:** Aibek E. Mirrakhimov, Alaa M. Ali, Carolyn Stroncek

**Affiliations:** ^1^Saint Joseph Hospital, Department of Internal Medicine, 2900 North Lake Shore, Chicago, IL 60657, USA; ^2^Saint Mary and Elizabeth Medical Center, Department of Emergency Medicine, 2233 West Division Street, Chicago, IL 60622, USA

## Abstract

Chest pain is one of the most common reasons for emergency department visits. Emergency medicine doctors should focus their initial assessment on patients' stability. History, physical examination, and ancillary testing should exclude serious causes such as acute coronary syndrome, acute aortic syndromes, pulmonary embolism, pneumothorax, esophageal perforation, and rupture as well as pericardial tamponade. Young age should not be used alone as a predictor of a benign condition. Below we present a case of a 24-year-old female who was found to have ascending aortic dissection and was sent for emergent surgery.

## 1. Introduction

Chest pain or chest discomfort is one of the most common reasons for emergency department (ED) visits in USA [1]. Chest pain is a very nonspecific symptom and has a huge differential diagnosis including some benign conditions such as musculoskeletal chest pain, esophageal spasm, and gastroesophageal reflux disease as well as more serious conditions such as acute coronary syndrome (ACS). Emergency medicine doctors and acute care providers who are on the frontiers of initial management and patient triage face everyday challenges in evaluating these patients. The most critical task of evaluation is to rule out potentially life-threatening causes of chest pain which include ACS, acute aortic syndrome, esophageal rupture, pulmonary embolism (PE), pneumothorax, pericardial effusion, and tamponade [2–4]. Of note, the aforementioned life-threatening causes of chest pain are relatively rare which makes a thorough history and physical examination an essential part of clinical triage and work-up. Clinicians should pay special attention to patient's age, gender, smoking history, prior history of similar chest pain, family history of cardiovascular and pulmonary diseases, presence of any alleviating or aggravating factors, quality of the chest pain/discomfort, presence of pain radiation, and presence of associated symptoms such as diaphoresis, nausea, vomiting, shortness of breath, dizziness, syncope, and abdominal pain. Physical examination should focus on airway, breathing, and circulation as in every patient in the emergency department as well as evaluating the presence of reproducible chest pain, cardiovascular examination including assessment of peripheral pulses, pulmonary examination, and abdominal examination at its minimum.

A common bias which is encountered in medicine is that young patients and especially females do not have life-threatening causes of chest pain. While this approach or belief will probably be right in the majority of times, however, an acute care provider should aim to rule out dangerous etiologies first with a good history, physical examination, 12-lead electrocardiogram (EKG), and a chest X-Ray (CXR). Further work-up and management should be based on the clinical impression of the clinician and the results of the initial diagnostic work-up. Below we present a case of a young female with a life-threatening entity.

## 2. Case Presentation

A 24-year-old female presented to our ED with a six-hour duration history midsternal chest pain of pleuritic quality (rated as 8 out of 10 on a pain scale) radiating to her neck and jaw. The chest pain was continuous after its onset, not related to rest or exertion with no reported alleviating or aggravating factors. The patient denied any history chest trauma. Family history was negative for premature cardiovascular disease or early death.

Review of systems was otherwise negative for cough, sweating, nausea, vomiting, abdominal pain, and diarrhea. The patient was a never smoker, drank alcohol socially, and did not use any recreational drugs. The patient had a history of bronchial asthma which was well controlled. The patient used albuterol as needed and did not report using oral contraceptive pills or other medications. The patient did not report any previous surgeries, immobilization, long distance travel, and personal or family history of blood clots or cancer.

Blood pressure was 133/56 mm Hg, heart rate was 70, respiratory rate was 18, oxygen saturation was 100% on room air, and temperature was 97.6 F (36.4 C). On a physical examination, the patient was in moderate distress due to chest pain, with no jugular venous distention, normal pulmonary examination with good bilateral breath sounds, minimally reproducible chest pain on palpation, and normal heart rate and normal heart sounds with no murmurs, rubs, or gallops and equal bilateral pulses. Abdominal and neurological examination was unremarkable. There was no extremity swelling or erythema. However, pectus excavatum or “sunken chest” was noted by a physical examination.

**Figure 1:**
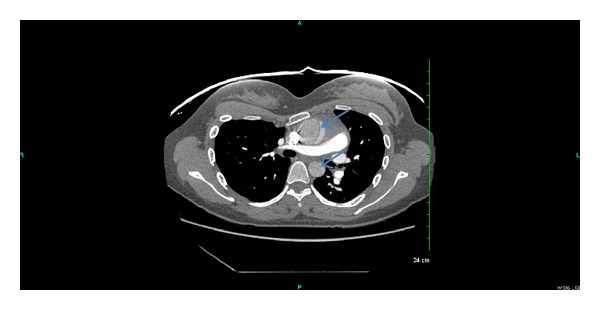
CT chest with IV contrast showing intimal flap (arrows) in both ascending and descending aortae.

12-lead EKG was done which showed normal sinus rhythm, normal axis, normal rate, normal intervals, and no evidence of T wave and ST segment abnormalities on 2 separate occasions 3 hours apart. CXR did not show pneumothorax, pneumonia, esophageal rupture, or perforation and was read as normal. Troponin was negative. The patient was deemed to be a low pretest probability for PE according to the Wells score and D-dimer was ordered [5, 6]. D-dimer was found to be elevated at 3041 ng/mL (normal range <500 ng/mL). Computed tomography (CT) of the chest with intravenous (IV) contrast was ordered. CT chest with IV contrast showed type A Stanford dissection involving the ascending aorta involving the aortic arch and great vessels (please see Figure 1) [2]. The patient was immediately transferred to a tertiary center for emergent aneurysm repair surgery. The patient surgery and inpatient stay were uneventful with return to a baseline functional level.

Our patient was tested positive for type IV Ehlers-Danlos syndrome. Ehlers-Danlos type IV syndrome is a genetic disease (typically autosomal-dominant one) with predisposition rupture of vasculature, intestine, and uterus [7]. Given the fact that there was no family history of Ehlers-Danlos syndrome in the family, it is possible that this could represent a de novo mutation [7].

## 3. Conclusion

Patients presented to the ED with chest pain represent a significant challenge to acute health care providers. Clinicians dealing with acute presentation of chest pain must exclude serious conditions first such as ACS, aortic dissection, pneumothorax, esophageal perforation, PE, and pericardial tamponade [2–4]. Clinical examination should focus on the airway, breathing, circulation, and presence of distal pulses in particular. In our patient, a pectus excavatum was a clinical clue to underlying aortic problem. Patients with ascending aortic dissection or Stanford type A should undergo emergent cardiovascular repair. Patients with descending aortic dissection or Stanford type B should be treated with medical therapy such as pain control, control of blood pressure, and heart rate [2]. Certain patients with Stanford type B dissection should undergo invasive management (surgery or stenting in selected cases) if their pain is not controlled and there is an evidence of end organ ischemia (e.g., limb ischemia, bowel ischemia, etc.), propagation, or expansion of the dissection as well as aortic rupture [2]. In terms of the disposition, all patients with aortic dissection should eventually go to intensive care unit (ICU) from ED. However, patients with Stanford type A aortic dissection should go to surgery first. It is also important to note that patients with Stanford type A aortic dissection should be transferred to a center experienced with aortic dissection repair whenever possible.
